# Laryngeal Mask Airway-Guided Fiberoptic Tracheal Intubation After Traumatic Failed Intubation in a Neonate With Severe Arthrogryposis Multiplex Congenita: A Case Report

**DOI:** 10.7759/cureus.114459

**Published:** 2026-08-13

**Authors:** Farah AlShammari, Khaled Ahmed, Ihab M Ali, Jahed J Albarqi, Mohammad Riyad, Khaled Sarhan, Thamer AlKharji, Fatemah Qasem

**Affiliations:** 1 Anesthesiology, Kuwait Institute for Medical Specialization, Kuwait City, KWT; 2 Pediatric Anesthesiology, New Maternity Hospital, Kuwait City, KWT; 3 Anesthesiology and ICU, Maternity Hospital, Kuwait City, KWT

**Keywords:** arthrogryposis multiplex congenita, congenital abnormalities, difficult airway, fiberoptic intubation, neonatal airway, prolonged laryngeal mask airway, rare disease, supraglottic airway

## Abstract

Airway management in neonates with arthrogryposis multiplex congenita (AMC) presents extreme challenges due to craniofacial abnormalities, cervical rigidity, limited mouth opening, and restrictive chest wall mechanics. These patients are at increased risk of failed intubation and perioperative respiratory complications, as airway trauma may rapidly develop, causing airway edema and compromising oxygenation. Early recognition and individualized airway planning are essential to avoid repeated traumatic airway manipulation and its associated complications.

In our case, a full-term male neonate with prenatally diagnosed AMC developed severe respiratory distress immediately after birth due to restrictive lungs. Multiple direct and indirect laryngoscopy attempts failed, resulting in significant airway edema and bleeding. Instead of pursuing repeated traumatic attempts, a supraglottic airway device (laryngeal mask airway (LMA)) was inserted to maintain ventilation and oxygenation. The LMA remained in situ continuously for 72 hours while airway edema subsided under intensive care monitoring. Once airway conditions improved, fiberoptic bronchoscopy-guided tracheal intubation was successfully performed through the LMA. The ENT team remained immediately available for emergency surgical airway throughout the procedure. No airway complications or pressure-related mucosal injury attributable to prolonged LMA use were observed in our case.

In selected neonates with traumatic difficult airways, prolonged LMA use may safely preserve oxygenation while allowing time for careful planning of definitive airway management. Fiberoptic-guided intubation through the LMA represents a practical and minimally traumatic approach.

## Introduction

Arthrogryposis multiplex congenita (AMC) comprises a group of congenital disorders with a prevalence between 1/3,000 and 1/5,200 live births. It is characterized by multiple non-progressive joint contractures involving at least two body regions [1]. Although the underlying etiologies vary, which can be due to extrinsic factors (oligohydramnios) or primary neurogenic or myopathic conditions of the fetus, decreased fetal movement results in joint contractures and musculoskeletal abnormalities [2]. Airway management in these patients may be particularly challenging because of micrognathia, cleft palate, temporomandibular joint restriction, cervical spine rigidity, reduced mouth opening, and associated craniofacial abnormalities. Respiratory compromise is further exacerbated by restrictive thoracic deformities and poor chest wall compliance, making effective ventilation difficult during airway management [3,4]. Current pediatric difficult airway guidelines emphasize limiting repeated laryngoscopy attempts, prioritizing oxygenation, early use of supraglottic airway devices, and involving experienced multidisciplinary teams when managing anticipated difficult airways [5-7]. Although laryngeal mask airways (LMAs) are well established as rescue devices in pediatric difficult airway management, prolonged continuous use as a bridge to delayed fiberoptic intubation has rarely been described in neonates with AMC. We report successful prolonged LMA ventilation for 72 hours followed by definitive fiberoptic intubation in a neonate with AMC after traumatic failed intubation attempts.

## Case presentation

A full-term male neonate weighing 3.17 kg at 40 weeks of gestation was prenatally diagnosed with AMC based on fetal ultrasonography and genetic evaluation. The mother was admitted to the labor ward for planned vaginal delivery; however, an emergency cesarean section was performed because of a non-reassuring fetal heart rate tracing. The neonate had Apgar scores of 6 and 7 at one and five minutes, respectively.

**Figure 1:**
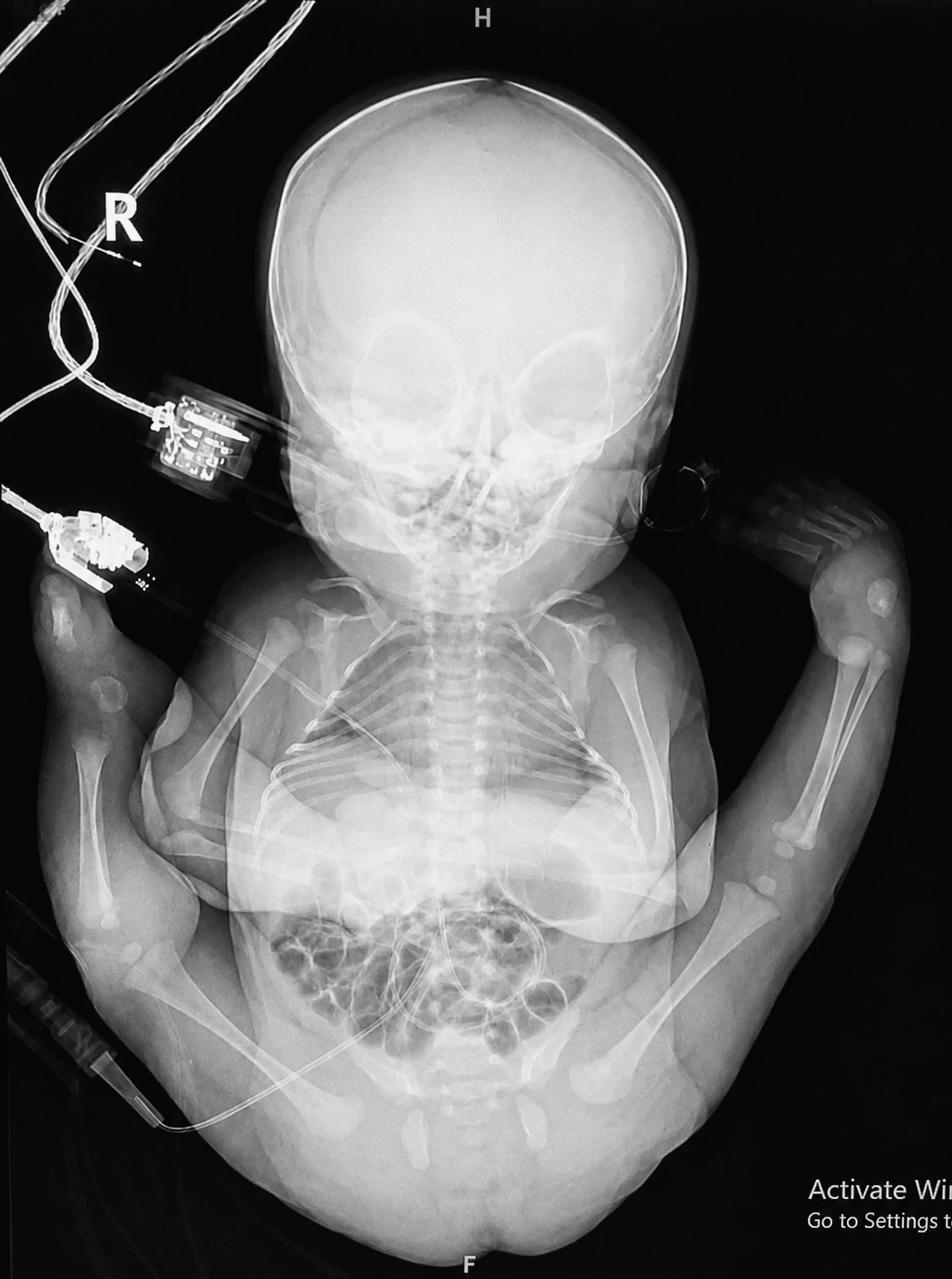
Preoperative whole-body anteroposterior radiograph of the neonate showing multiple congenital musculoskeletal deformities consistent with arthrogryposis multiplex congenita (AMC), including severe limb contractures and craniofacial abnormalities, indicative of a predicted difficult airway.

Immediately after birth, the infant developed significant respiratory distress requiring urgent ventilatory support. Airway examination revealed markedly limited jaw movement and mouth opening, severe neck stiffness, and profound restriction of cervical extension. Physical examination also demonstrated generalized joint contractures and a rigid, poorly compliant thoracic cage (Figure 1), suggesting both a predicted difficult airway and impaired respiratory mechanics. Four attempts at endotracheal intubation using direct and indirect laryngoscopy with a Miller 0 blade were unsuccessful. Initial attempts were performed by the neonatologist, followed by two experienced pediatric anesthesiologists, with failure attributed to inadequate visualization of the laryngeal structures. Repeated airway instrumentation resulted in mucosal bleeding and progressive laryngeal edema, further compromising glottic visualization. Despite the repeated attempts, adequate oxygenation was maintained, with oxygen saturation consistently above 92%. Given the increasing airway trauma and worsening edema, it was considered that further attempts at direct tracheal intubation would likely increase airway injury without significantly improving the probability of successful intubation. The airway management strategy was therefore modified, and an Ambu LMA AuraOnce size 1 (Xiamen, Fujian, China) was inserted to establish a secure supraglottic airway and maintain ventilation and oxygenation. Adequate ventilation was confirmed by the presence of an appropriate capnographic waveform, and the patient was subsequently transferred to the neonatal intensive care unit (NICU).

Following multidisciplinary discussion, the decision was made to defer definitive tracheal intubation and allow the airway edema to resolve before attempting further airway instrumentation. The LMA was therefore maintained in situ for approximately 72 hours under close NICU observation. During this period, the patient was managed with pressure-controlled mechanical ventilation. Initial ventilator settings included a peak inspiratory pressure of 16 cmH₂O, positive end-expiratory pressure (PEEP) of 5 cmH₂O, respiratory rate of 35 breaths/min, inspiratory time of 0.4 s, and FiO₂ ranging from 0.25 to 0.30, adjusted to maintain oxygen saturation between 92% and 97%. Enteral nutrition was provided via a nasogastric tube throughout the 72-hour period of LMA placement. Feeding was initiated cautiously and advanced according to the patient's clinical condition and tolerance. The patient tolerated enteral feeding without episodes of regurgitation, vomiting, aspiration, abdominal distension, or other evidence of feeding intolerance. Intermittent gastric decompression was performed as clinically indicated to minimize gastric insufflation. Airway edema was managed conservatively to allow spontaneous resolution. Dexamethasone was administered at a dose of 0.25 mg/kg every eight hours for a total of three doses. Throughout this period, the patient remained clinically stable, with no evidence of airway compromise or feeding-related complications.

**Figure 2:**
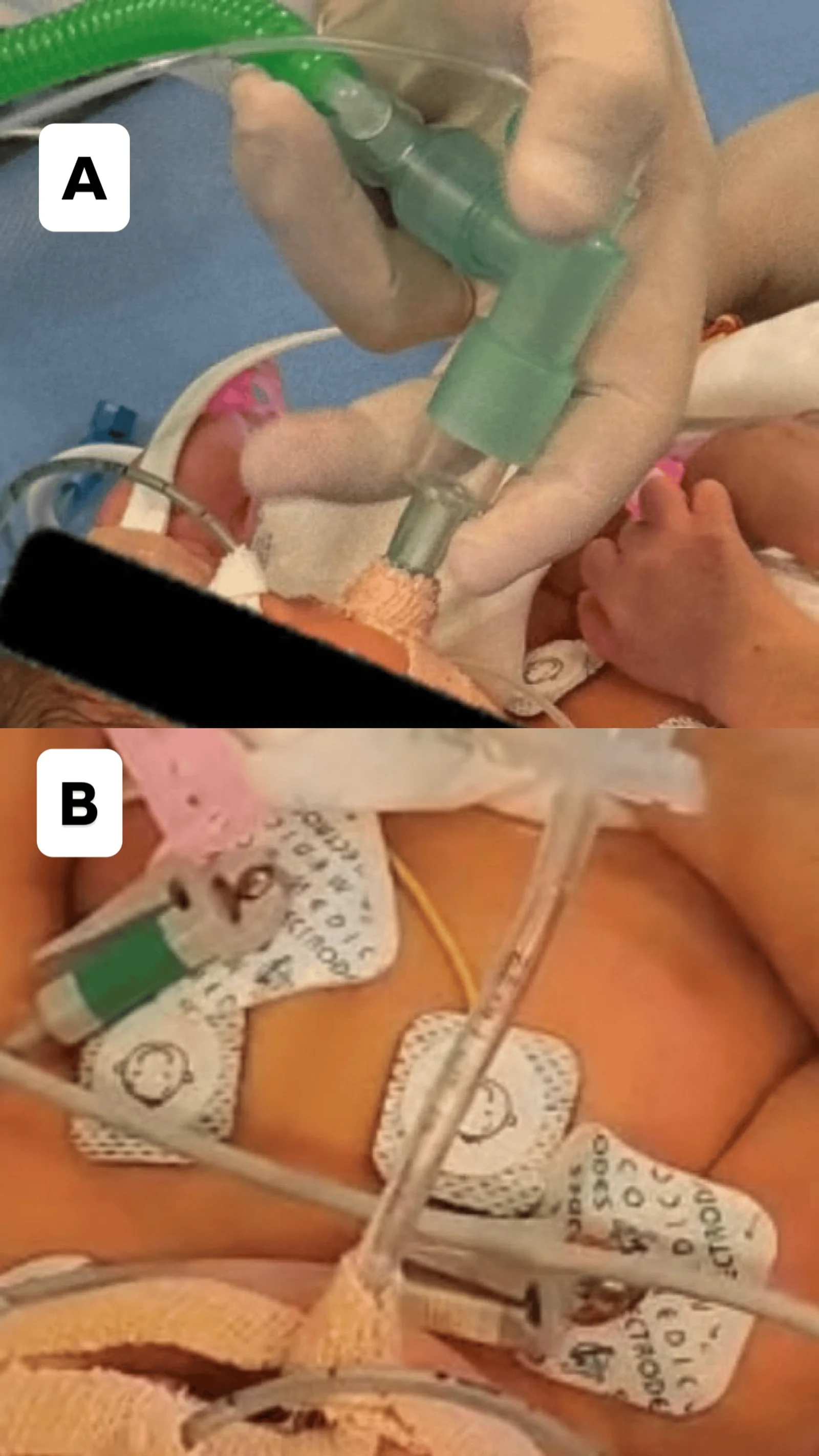
Fiberoptic-guided tracheal intubation through a laryngeal mask airway (LMA) in a neonate with a traumatic difficult airway. (A) The LMA provided effective ventilation and served as a conduit for fiberoptic bronchoscopy. (B) Successful tracheal intubation was achieved over the fiberoptic bronchoscope, with the endotracheal tube securely positioned after LMA removal.

On day 4 of life, the patient was transferred to the operating theater for definitive airway management. A multidisciplinary team comprising pediatric anesthesiologists, neonatologists, and respiratory therapists was assembled, with the otolaryngology (ENT) team immediately available throughout the procedure should a surgical airway become necessary. Anesthetic induction was achieved with propofol 5 mg, fentanyl 5 μg, and glycopyrrolate 15 μg, without administration of a neuromuscular blocking agent. A flexible fiberoptic bronchoscope (single-use, 2.6 mm outer diameter) was then advanced through the LMA, allowing direct visualization and assessment of the glottic opening. By this time, the previously observed airway edema had substantially subsided, resulting in markedly improved visualization of the laryngeal structures compared with the initial intubation attempts. An uncuffed 3.5 mm endotracheal tube was subsequently advanced through the LMA under direct flexible fiberoptic bronchoscopic guidance and successfully positioned within the trachea (Figure 2 and Video 1). Following successful intubation, inspection of the upper airway revealed no evidence of tongue edema, mucosal ulceration, pressure necrosis, or other pressure-related complications attributable to the prolonged placement of the LMA.

**Video 1:** Fiberoptic-guided tracheal intubation through an LMA in a neonate with a traumatic difficult airway. The video demonstrates bronchoscopic visualization of the glottis, advancement into the trachea with identification of the carina, railroading of the endotracheal tube over the bronchoscope, and successful tracheal intubation while maintaining oxygenation throughout the procedure. LMA: laryngeal mask airway

## Discussion

Our case highlights several important principles in the management of the anticipated neonatal difficult airway. Rather than persisting with repeated laryngoscopy after significant airway trauma had already occurred, the airway management strategy was individualized to prioritize oxygenation over immediate tracheal intubation. This approach is consistent with current pediatric difficult airway guidelines, which emphasize limiting repeated laryngoscopy attempts because each additional attempt increases the risk of airway edema, bleeding, hypoxemia, and complete airway failure [5,6].

Although LMAs are widely recommended as rescue devices during failed intubation, they are generally intended for short-term airway management during anesthesia [5,6]. Prolonged use of the LMA in neonates has rarely been reported, as it raises concerns regarding potential complications including mucosal ischemia, tongue edema, aspiration, inadequate ventilation, gastric insufflation, and pressure necrosis. Fernández-Jurado and Fernández-Baena described successful ventilatory support through an LMA for 44 hours in a preterm neonate after failed intubation without airway complications [8]. Ames et al. later reported successful long-term LMA use in a neonate as a bridge to definitive airway management [9]. More recently, Lorente et al. described a term neonate managed with an LMA for seven consecutive days; although ventilation remained adequate, prolonged placement resulted in significant pharyngeal edema and mucosal injury, complicating subsequent tracheal intubation [10]. In contrast, our patient underwent prolonged LMA-assisted ventilation following airway trauma, after which definitive airway control was successfully achieved by fiberoptic-guided tracheal intubation through the LMA. To our knowledge, reports describing this combination of prolonged LMA use and delayed fiberoptic intubation in a neonatal traumatic airway are exceedingly rare.

One of the main concerns regarding prolonged use of LMA is the increased risk of pulmonary aspiration. Unlike an endotracheal tube, LMA is not a definitive airway to protect against regurgitated gastric contents, given the immature lower esophageal sphincter, relatively large gastric volumes, and reduced physiological reserve in neonates. However, several measures were taken to mitigate this risk while the LMA served as a conduit to definitive airway management. The patient was monitored in the intensive care unit. Gastric decompression was maintained using a nasogastric tube. Airway pressures during ventilation were kept as low as possible to minimize gastric insufflation, while ensuring adequate oxygenation and ventilation. Regular suctioning of oral and pharyngeal secretions was performed to reduce secretion pooling above the LMA. Throughout the period of LMA ventilation, there were no clinical signs of regurgitation or aspiration, and oxygenation remained stable.

The decision to continue ventilation through the LMA represented a careful balance between competing risks. Repeated direct laryngoscopy in the setting of traumatic airway injury was considered likely to worsen edema, bleeding, and airway distortion, potentially converting a ventilatable airway into a "cannot intubate, cannot oxygenate" scenario. In contrast, the LMA provided reliable oxygenation while allowing time for stabilization and planning of a controlled fiberoptic intubation.

Although prolonged LMA use should not replace definitive airway management, our experience suggests that, in carefully selected neonatal patients under intensive monitoring, it can serve as a safe temporary bridge when the risks of repeated intubation attempts outweigh the potential aspiration risk. The absence of aspiration-related complications in our patient supports the feasibility of this strategy, although careful patient selection and vigilant monitoring remain essential.

Finally, dexamethasone was used in our case as an adjunctive treatment to reduce airway edema caused by repeated traumatic intubation attempts while effective oxygenation and ventilation were maintained through the LMA. Although corticosteroids should not delay definitive airway management, they may promote resolution of mucosal inflammation. An evidence-based regimen of dexamethasone 0.25 mg/kg intravenously every eight hours for three doses has been demonstrated to reduce post-extubation stridor and the need for reintubation. While these data are derived from the prevention of post-extubation laryngeal edema, the same anti-inflammatory mechanism supports its use as an adjunct in selected cases of traumatic neonatal airway injury, as in our patient [11].

## Conclusions

This case demonstrates that prolonged LMA ventilation may serve as a safe bridge to definitive airway management in selected neonates with anticipated difficult airways. Individualizing the neonatal difficult airway algorithm, prioritizing oxygenation, and avoiding repeated traumatic intubation attempts may improve patient safety.
